# Innate lymphotoxin receptor mediated signaling promotes HSV-1 associated neuroinflammation and viral replication

**DOI:** 10.1038/srep10406

**Published:** 2015-05-20

**Authors:** Yong Liang, Kaiting Yang, Jingya Guo, Joanna Wroblewska, Yang-Xin Fu, Hua Peng

**Affiliations:** 1 Key Laboratory of Infection and Immunity, Institute of Biophysics, Chinese Academy of Sciences, Beijing 100101, China; 2 University of Chinese Academy of Sciences, Beijing 100049, China; 3 Department of Pathology and Committee on Immunology, University of Chicago, Chicago, Illinois 60637, USA

## Abstract

Host anti-viral innate immunity plays important roles in the defense against HSV-1 infection. In this study, we find an unexpected role for innate LT/LIGHT signaling in promoting HSV-1 replication and virus induced inflammation in immunocompromised mice. Using a model of footpad HSV-1 infection in Rag1^–/–^ mice, we observed that blocking LT/LIGHT signaling with LTβR-Ig could significantly delay disease progression and extend the survival of infected mice. LTβR-Ig treatment reduced late proinflammatory cytokine release in the serum and nervous tissue, and inhibited chemokine expression and inflammatory cells infiltration in the dorsal root ganglia (DRG). Intriguingly, LTβR-Ig treatment restricted HSV-1 replication in the DRG but not the footpad. These findings demonstrate a critical role for LT/LIGHT signaling in modulating innate inflammation and promoting HSV-1 replication in the nervous system, and suggest a new target for treatment of virus-induced adverse immune response and control of severe HSV-1 infection.

Herpes simplex virus type 1 (HSV-1) is a ubiquitous human pathogen causing acute, latent, reactive, and persistent infections^1^. HSV-1 invades the human host through the oral mucosa and establishes lifelong latency in the trigeminal ganglion. Although HSV-1 infection usually causes only mild clinical disease such as herpes labialis or “cold sores”, it could lead to lethal herpes simplex encephalitis (HSE) in neonates or immunocompromised individuals^2^. HSE is associated with active viral replication and vigorous inflammation in the central nervous system (CNS). Although innate cytokines, such as type I IFN and TNF, are required for HSV-1 control, excessive innate inflammatory response could be harmful to the host^3^,^4^,^5^. Depletion of macrophage or neutrophil improved the survival of infected 129S6 mice, which are susceptible to HSE^6^. TLR2 has been reported to promote HSV-induced lethal encephalitis through mediating innate cytokine response^7^. A more thorough understanding of the signaling pathways and mechanisms that regulate innate anti-viral response to HSV-1, as well as maladaptive inflammation, would be informative in the development of clinical treatments for HSV-1 associated diseases.

Lymphotoxin-beta receptor (LTβR), a member of the TNFR superfamily, binds to two ligands: Lymphotoxin-α1β2 (LT) and LIGHT (homologous to LT, exhibits inducible expression, and competes with HSV glycoprotein D for HVEM, a receptor expressed by T lymphocytes). LT-LTβR signaling-induced chemokine and cell migration is required for the maintenance of secondary lymphoid tissue structure^8^,^9^. The development and maintenance of marginal zone macrophages, follicular dendritic cells and the organized structure in the spleen by LT signaling is important to the production of type 1 IFN, IgG and CD8+ T cell response against viral infections, such as lymphocytic choriomeningitis virus (LCMV) and vesicular stomatitis virus^10^,^11^,^12^,^13^. In addition, LT-LTβR signaling could directly promote type 1 IFN expression against mouse cytomegalovirus (MCMV) infection in the spleen^14^. LT signaling has been shown to play a key role in adaptive immunity against HSV-1. HSV-1 specific IgG responses are impaired in LTα^–/–^ mice when immunized with UV-inactivated virus^15^. LTα^–/–^ mice have impaired HSV-1 specific T cell effector function and fail to control viral infection of the central nervous system^16^. LIGHT is mainly expressed on immature dendritic cells (DC) and activated T cells. It was first found to be a co-stimulator of T cells functioning through the HVEM receptor^17^,^18^,^19^. LIGHT interaction with LTβR can upregulate proinflammatory chemokines and adhesion molecules, which recruit and activate immune cells^20^,^21^. There is no report yet about the function of this molecule in HSV-1 infection. Overall, previous studies in immunocompetent mice suggest that LT-LTβR signaling may play a protective role against HSV-1 infection through regulating adaptive immunity. To our surprise, Rag1^–/–^ mice treated with blockade of LT/LIGHT signaling showed increased resistance to HSV-1 infection: delayed development of lesions and increased survival. Our study suggests that innate LT/LIGHT signaling may be exploited to promote HSV-1 replication and virus-induced neuroinflammation in immunocompromised mice.

## Results

### LT/LIGHT signaling contributes to HSV-1 induced morbidity and mortality in Rag1^–/–^ mice

**Figure 1:**
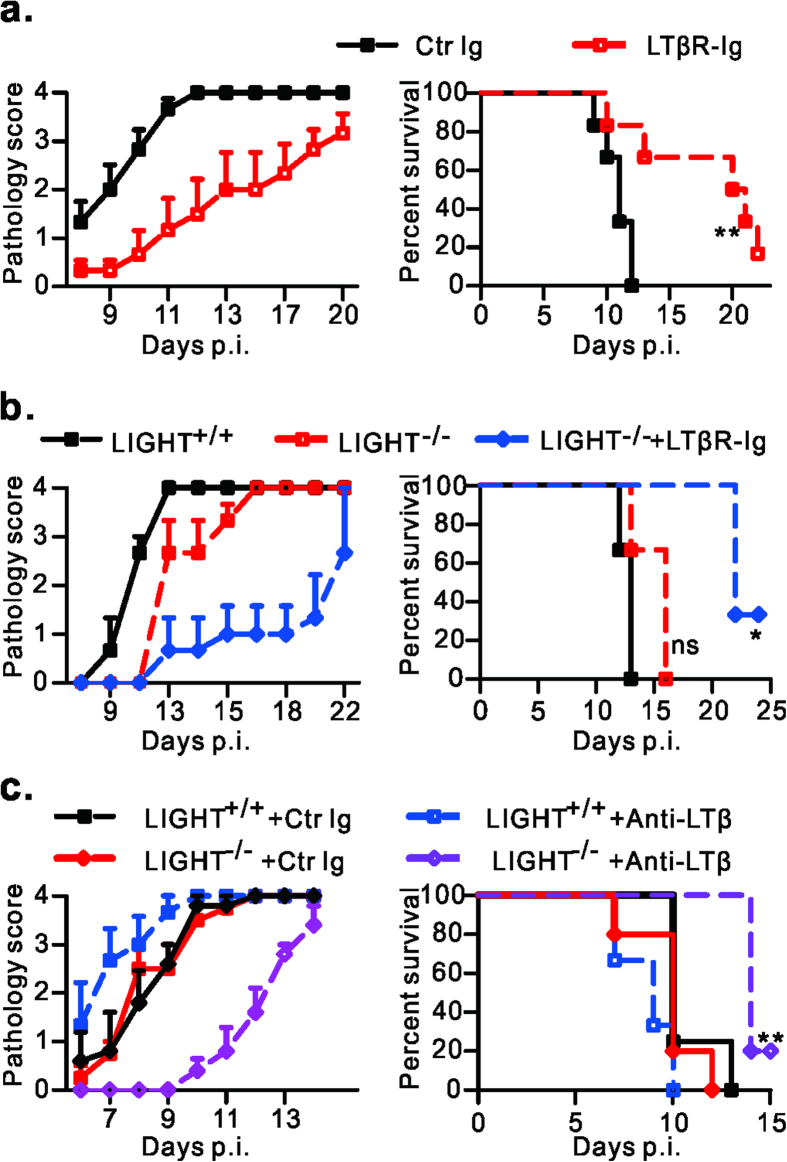
Blockade of LT/LIGHT inhibits disease progression and extends the survival of Rag1^–/–^ mice after HSV-1 infection. (**a**) Rag1^–/–^ mice (n = 6/group) were infected with 2 × 10^6^ pfu of HSV-1 and treated with 100 ug of LTβR-Ig or control protein on day -1 and day 5 p.i. (**b**, **c**) Rag1^–/–^ LIGHT^+/+^ or Rag^–/–^LIGHT^–/–^ mice (n = 3 to 5/group) were infected with 2 × 10^6^ pfu of HSV-1 and treated with LTβR-Ig (**b**), anti-LTβ (**c**) or control protein as indicated. Anti-LTβ was administrated at 250 ug/mice every 3 days starting from day -1. Mice were observed for the development of skin lesion, general behavioral changes and survival. The severity of morbidity was scored as follows: 0+, normal; 1+, footpad swell; 2+, show ulceration in the skin of thigh; 3+, tail incline to one side or paralysis; 4+, moribund mice which were euthanized. The pathology score at the indicated time points are shown. Ctr Ig, control protein. Data are representative of more than three (**a**) or two (**b**, **c**) independent experiments. Statistical analysis for survival data was by log rank test.

LT-LTβR signaling has been shown mostly to play a key role in adaptive effector function against HSV-1 and other pathogens^11^,^12^,^16^. This signaling pathway is also involved in regulating innate immunity against infection of cytomegalovirus, a member of Herpesviridae family^14^,^22^. To explore the role of LTβR signaling in the innate response to HSV-1 infection, HSV-1 infected Rag1^–/–^ mice were treated at day -1 and 5 with soluble LTβR-Ig, which blocks the LT/LIGHT interaction with LTβR^23^,^24^. Unexpectedly, LTβR-Ig treatment greatly inhibited the disease progression of infected Rag1^–/–^ mice. Control Ig treated mice exhibited skin lesions starting as early as on day 8. Mice treated with LTβR-Ig were asymptomatic until day 11 (Fig. 1a). Notably, control animals died at about 10 days post infection (p.i.), while LTβR-Ig treatment significantly (P = 0.0098) extended the survival of infected mice to about day 20 p.i. (Fig. 1a). These data suggest that LT/LIGHT signaling promotes HSV-1 associated pathogenesis and death in immunocompromised mice.

LTβR-Ig binds to two ligands of LTβR: LTαβ and LIGHT. To figure out which LTβR ligand plays a role in promoting HSV-1 associated pathogenesis, Rag1^–/–^ or Rag^–/–^LIGHT^–/–^ mice were infected and treated with LTβR-Ig, anti-LTβ antibody or control protein. The progression of disease in Rag^–/–^LIGHT^–/–^ mice was similar to Rag1^–/–^ mice. Furthermore, LTβR-Ig treatment could still increase the resistance of Rag^–/–^LIGHT^–/–^ mice to HSV-1 infection (Fig. 1b). These results suggest that LIGHT is not essential to drive pathogenesis. Blockade of the interaction of LT-LTβR with anti-LTβ antibody in Rag1^–/–^ mice showed no protective effect (Fig. 1c), raising the possibility that blocking both ligands is required for enhancing mouse resistance to HSV-1 infection. Indeed, treatment with anti-LTβ alleviated disease symptoms and extended the survival of Rag^–/–^LIGHT^–/–^ mice (Fig. 1c). Deficiency for either LTβ or LIGHT could not recapitulate the protective effect of LTβR-Ig against HSV-1, indicating a redundant or compensatory role of LT and LIGHT in the HSV-1 infection induced pathogenesis (Fig.1b,c).

### LT/LIGHT signaling stimulates late systemic and local proinflammatory cytokine release induced by HSV-1 infection

**Figure 2:**
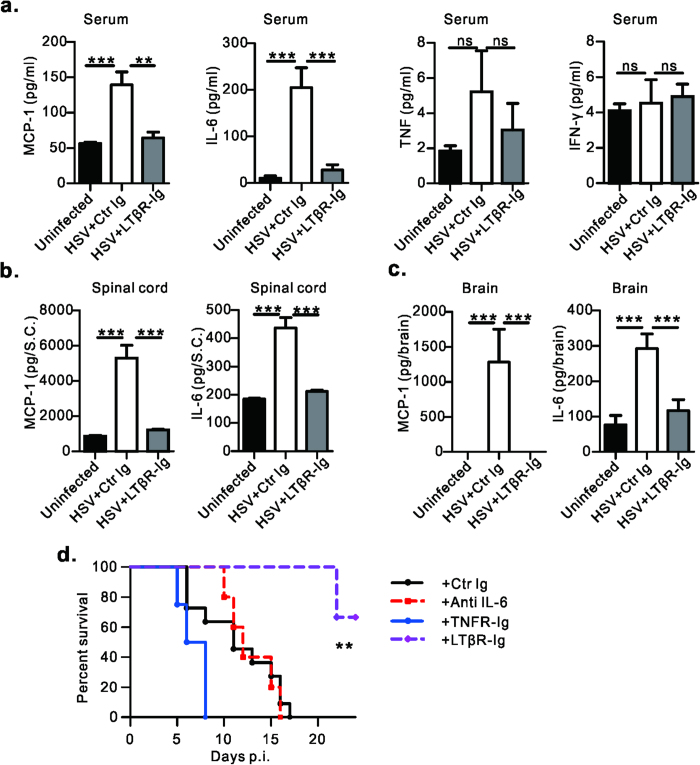
Blockade of LT/LIGHT inhibits late systemic and local proinflammatory cytokines release induced by HSV-1 infection. (**a**) Rag1^–/–^ mice (n = 7 to 9/group) were injected with 1 × 10^7^ pfu of HSV-1 and treated with LTβR-Ig or control protein as Fig. 1A. On day 7 p.i., cytokine levels of IL-6, MCP-1, TNF and IFN-γ in the serum were determined by CBA. Data are pooled from two independent experiments. (**b**, **c**) On day 7 p.i., cytokine levels in the homogenate of spinal cord (**b**) and brain (**c**) were determined. n = 4 to 5/group. Data are representative of two independent experiments. (**d**) Rag1^–/–^ mice (n = 4 to 11/group) were infected with 2 × 10^6^ pfu of HSV-1 and treated with indicated antibody or protein to block *in vivo* cytokine signaling. TNFR-Ig or LTβR-Ig was administrated at 100 ug/mice on day-1 and day 5 p.i.; Anti-IL-6 was administrated at 400 ug/mice every 3 days starting from day -1. Data are representative of two independent experiments. Uninfected, mice without infection; HSV+ Ctr Ig, infected mice with control protein treatment; HSV+ LTβR-Ig, infected mice with LTβR-Ig treatment. Statistical analysis for **a**, **b**, **c**. was by unpaired t test. Error bar represents SEM, *p < 0.05, **p < 0.01, ***p < 0.001; ns, no significant difference. **d**. was by log rank test.

HSV-1 more frequently reactivates in immunocompromised hosts, causing intense inflammation in the mucosa and brain tissues. To explore the effect of LT/LIGHT signaling in HSV-1 -induced inflammation, Cytometric Bead Array was utilized to determine the serum cytokine profile following infection. Rag1^–/–^ mice injected subcutaneously with HSV-1 in the footpad showed a biphasic proinflammatory cytokine response. At early time points, such as 24 hours p.i., increased levels of IL-6, MCP-1 and TNF were detected in serum (Supplemental Fig. 1a). Cytokine levels were reduced at 72 hours p.i. (data not show). On day 7 p.i., Rag1^–/–^ mice started exhibiting signs of illness and body weight loss. Though the level of IFN-γ, IL-10 and IL-12 were very low or undetectable at this time point (data now show), significantly elevated levels of other proinflammatory cytokines were detected in the serum, especially IL-6 and MCP-1 and a trending increase in TNF (Fig. 2a). High levels of IL-6 and MCP-1 were also found in spinal cord and brain indicating that viral infection had induced intense inflammatory response in CNS (Fig. 2b,c). These kinetics suggest that the second wave of cytokine responses might play some role in the HSV-1 associated pathogenesis. Interestingly, LTβR-Ig treatment at day -1 and 5 substantially reduced the level of proinflammatory cytokines in the serum at day 7 p.i. (Fig. 2a). LTβR-Ig treatment also reduced the production of both MCP-1 and IL-6 in the spinal cord (Fig. 2b) and brain (Fig. 2c). These data suggest that LT/LIGHT promotes the late wave of inflammation induced by HSV-1 infection.

To determine if the late wave of proinflammatory cytokine and mouse death were caused by HSV-1 invasion of CNS, viral titers were tracked in various tissues of moribund mice. Virus could be detected at the footpad infection site, brainstem and cerebellum. No virus was found in the spleen, lung or liver, suggesting no systemic viral dissemination occurred in Rag1^–/–^ mice (Supplemental Fig. 1b). To further confirm that HSV-1 spreading from sensory nerves to CNS is critical to infection-induced lethality in immunocompromised mice, sciatic nerve resection was performed one day before infection to block the transportation of HSV-1 from peripheral footpad to DRG and spinal cord. Indeed, this surgical operation greatly extended the survival of infected Rag1^–/–^ mice (Supplemental Fig. 1c). These data suggest that HSV-1 infection of the spinal cord and brain through the infected footpad acts to induce proinflammatory cytokine release, which is promoted by LT/LIGHT signaling.

To determine if the proinflammatory cytokines decreased by LTβR-Ig treatment contribute to disease progression, Rag1^–/–^ mice infected with HSV-1 were treated with blocking antibodies or blocking fusion proteins for various proinflammatory cytokines. Blockade of TNF resulted in slightly earlier death, and blockade of IL-6 had no effect (Fig. 2d). Neither treatment recapitulated the protective effect of LTβR-Ig. These data suggest that IL-6 and TNF induced by HSV-1 infection are not sufficient to promote disease progression.

### LT/LIGHT signaling promotes HSV-1 replication in DRG and the spinal cord, but not the footpad

**Figure 3:**
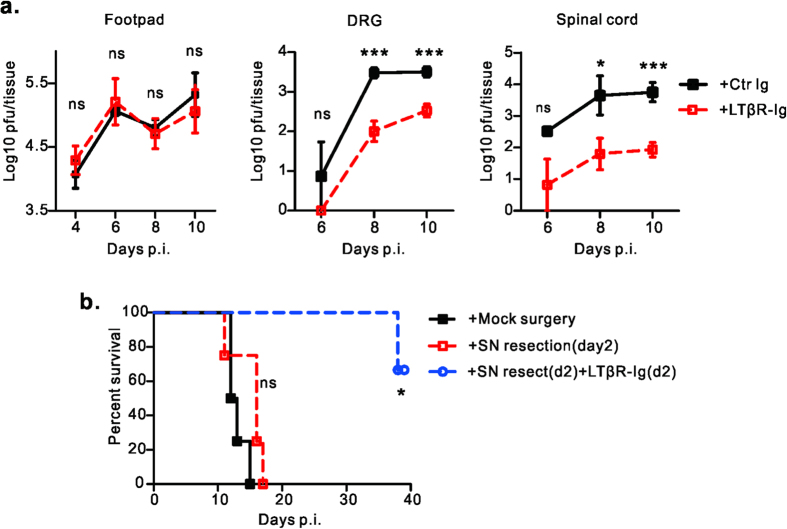
Blockade of LT/LIGHT signaling inhibits viral replication in nervous tissue. (**a**) Rag1^–/–^ mice (n = 4 to 7/group) were infected with 2 × 10^6^ pfu of HSV-1 and treated with either LTβR-Ig or control protein on day -1 and day 5 p.i.. At the indicated time points of figures, mice were euthanized. Footpad, DRG (L3, L4 and L5) and spinal cord were collected. Viral loads in different tissue homogenates were determined by plaque assay. (**b**) Rag1^–/–^ mice (n = 4 /group) were infected with 2 × 10^6^ pfu of HSV-1 via footpad injection. For group with sciatic nerve (SN) resection, one segment of the SN was removed on day 2 (d2) p.i., For the group with SN resection and blockade of LT/LIGHT, 100 ug/mice of LTβR-Ig was administrated on day 2 p.i. (after sciatic nerve resection) and day 8 p.i. Data are representative of two independent experiments. Statistical analysis for **a.** unpaired t test, Error bar represents SEM, *p < 0.05, **p < 0.01, ***p < 0.001; ns, no significant difference. **b.** log rank test.

To further determine how LTβR-Ig treatment protects Rag1^–/–^ mice from HSV-1, we examined the viral loads in tissues along the viral transportation pathway: from the infected footpad to dorsal root ganglia then to spinal cord^25^. There was no difference in HSV-1 viral load in the footpad between LTβR-Ig treated Rag1^–/–^ mice and control groups (Fig. 3a). High levels of HSV-1 were maintained in the footpad until day 10 p.i.. This was expected because Rag1^–/–^ mice can not clear HSV-1 without adaptive immunity^26^. However, viral loads in nervous tissues were reduced substantially in the LTβR-Ig treated group. On day 8 and day 10 p.i., while control mice had a high HSV-1 viral load in the lumbar DRGs which connect with sciatic nerve (L3, L4 and L5) (about 10^3^ pfu/mice), the viral load in the DRG of LTβR-Ig treated mice was notably lower than 10^2^ pfu (Fig. 3a). Similarly, about 100-fold lower viral loads were detected in the spinal cord of Rag1^–/–^ mice with LTβ R-Ig treatment than control group (Fig. 3a). We observed a rapid increase of HSV-1 viral titers in DRG through day 6 to day 8 in control mice but a greatly slower increase in LTβR-Ig treated mice (Fig. 3a). Together, these data suggest that LTβR-Ig treatment targets the DRG and inhibits the initial rapid HSV-1 replication in the infected sensory neurons.

To further prove this hypothesis, the sciatic nerve was resected on day 2 p.i., after the virus had established infection in the DRG^25^,^27^. The absence of further transportation of virus from the footpad to the ganglia following sciatic nerve resection did not alter the survival kinetics of Rag1^–/–^ mice compared to the mock operation group (Fig. 3b). This indicates that HSV-1 induced death resulted from viral amplification in the DRG and CNS but not the peripheral inoculation site. LTβR-Ig treatment starting from day 2 p.i., in the context of sciatic nerve resection, still prolonged the survival of Rag1^–/–^ mice, suggesting that LTβR-Ig treatment functions through inhibiting rapid HSV-1 replication in ganglia (Fig. 3b). These results suggest that LT/LIGHT signaling promotes HSV-1 replication in the DRG.

### LT/LIGHT signaling regulates innate immune cell recruitment into DRG, which might further stimulate HSV-1 infection

**Figure 4:**
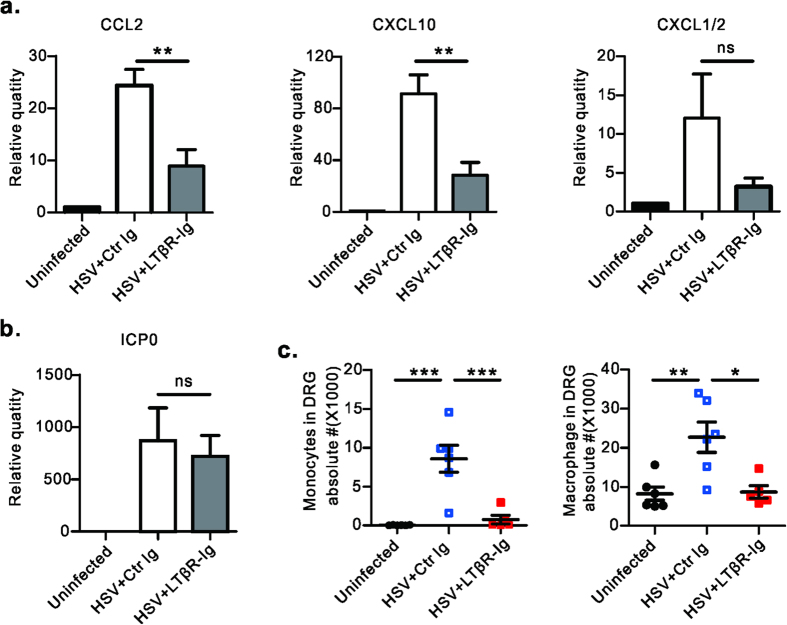
Blockade of LT/LIGHT inhibits chemokine expression and inflammatory cell infiltration into the DRG of infected Rag1^–/–^ mice. Rag1^–/–^ mice (n = 3 to 5/group) were infected with HSV-1 and treated with LTβR-Ig as Fig. 1a. Uninfected mice were chosen as the control group. On day 6 p.i., DRGs (L3, L4 and L5) were collected from euthanized mice. The mRNA level of various chemokines (CCL2, CXCL10 and CXCL1/2) (**a**) and viral ICP0 gene (**b**) were measured by real-time PCR. Data are representative of two independent experiments. (**c**) On day 8 p.i., innate immune cell subsets in DRG were determined by flow cytometry assay. Gate strategy: monocytes (CD45^+^CD11b^+^Ly6C^hi^Ly6G^middle^), macrophage (CD45^+^F4/80^+^). Data are pooled from two independent experiments, n = 5 to 6/group. Statistical analysis for **a**, **b**, **c** was by unpaired t test. Error bar represents SEM, *p < 0.05, **p < 0.01, ***p < 0.001; ns, no significant difference.

LT/LIGHT signaling can induce specific chemokines that recruit innate immune cells^9^,^20^,^21^. To determine if LT/LIGHT signaling affects HSV-1 replication in the DRG through regulating the immune microenvironment by recruited innate inflammatory cells, the effect of LT/LIGHT signaling in HSV-1 induced chemokines within the DRG was examined. On day 6 p.i., HSV-1 infection induced a high level of expression of CCL2 and CXCL10 within the ganglia, chemokines responsible for macrophage/monocyte recruitment (Fig. 4a). The mRNA level of neutrophil attracting CXCL1/2 was slightly but not significantly elevated following infection. Strikingly, LTβR-Ig treatment inhibited the expression of these chemokines (Fig. 4a), while the level of viral lytic associated gene ICP0 mRNA was not affected by LTβR-Ig treatment (Fig. 4b).

Next, we explored the effect of LT/LIGHT signaling on the recruitment of innate immune cells into DRG. On day 8 p.i., significant infiltration of inflammatory cells, consisting primarily of monocytes and macrophage were found in the DRG of Rag1^–/–^ mice (Fig. 4c). Consistent with the previous chemokine data, neutrophil numbers did not increase (Supplemental Fig. 2a). Impressively, LTβR-Ig treatment apparently inhibited macrophage and monocytes infiltration to the DRG on day 8 p.i. Mononuclear cell infiltration in DRG of infected mice was confirmed by H&E staining (supplemental Fig. 2b). IHC showed typical inflammation staining, where infected neuronal cells were surrounded by CD45+ mononuclear cells as highly dense foci within ganglia (supplemental Fig. 2b). Compared to control animals, LTβR-Ig treated mice had substantially reduced innate cell infiltration to the DRG (supplemental Fig. 2b).

## Discussion

HSV-1 infection in neonates and immunocompromised individuals can cause lethal encephalitis, with vigorous and detrimental inflammation in brain tissue. Previous studies show that LT signaling plays a protective role against HSV-1 infection. LTα^–/–^ mice had impaired virus-specific IgG and CD8+ T cell responses^15^,^16^. However, unlike wild type mice, severe HSV-1 related morbidity such as ulceration appeared at as early as day 6 after infection in LTα^–/–^ mice, at time point before the transition from innate to adaptive immunity. Because LT signaling was reported to be essential for type 1 IFN response,^14^,^22^ an anti-virus cytokine, we sought to determine if LT signaling could affect innate immunity to HSV-1 infection, using a model of HSV-1 footpad injection in Rag1^–/–^ mice which lack B and T cells.

Surprisingly, we found that blockade of LT/LIGHT signaling by LTβR-Ig fusion protein greatly delayed disease progression and extended the survival of infected Rag1^–/–^ mice (Fig. 1a). By using LTβR-Ig fusion protein which blocks both LT and LIGHT interacting with LTβR, as well as genetic models we showed that both LT and LIGHT contributed to promoting HSV-1 associated disease in Rag1^–/–^ mice (Fig. 1b,c). A similar redundant or compensatory role of LT and LIGHT during secondary lymphoid tissue organogenesis has been previously reported. Mice singly deficient for either LTβ or LIGHT retained mesenteric LN organogenesis, while an absence of LTβR or a deficiency of both LT and LIGHT resulted in more severe defects^28^. In addition, complementation with LIGHT interacting through LTβR could lead to splenic reconstitution in LT^–/–^ mice^29^.

HSV-1 frequently induces intense and detrimental inflammation in the mucosa and brain tissues of immunocompromised hosts. Using mouse model, we explored the effect of LT/LIGHT signaling in HSV-1-induced inflammation. In the mice with footpad injection of HSV-1, initial serum cytokines were found within hours after viral inoculation (Supplemental Fig. 1a). These cytokines may derived from infected epithelial cells, tissue resident macrophages and dendritic cells in the footpad. Virus then travel up to the dorsal root ganglia along the axon and take 5 to 7 days to lead to observable pathogenesis in the spinal cord^27^. Consistently, we found innate inflammatory cytokines were produced in the serum and CNS on day 7 p.i. and resulted in a late wave of cytokines in serum (Fig. 2a-c). It is reported that TLR-mediated innate cytokine responses are essential to control HSV-1 in the trigeminal ganglia and prevent virus spread to the brain^30^. However, innate cytokines such as CXCL10, MCP-1 and IL-6 are also associated with herpes encephalitis^7^,^31^. Thus, although essential for controlling viral infection, inflammatory cytokine could have a deleterious role in the CNS. We found that LTβR-Ig treatment could efficiently prevent late inflammation on day 7 p.i., which was correlated with less damage to the CNS of Rag1^–/–^ mice (Fig. 2a-c).

LT was previously reported to be essential to defense against invading viruses, which mostly due to its role in maintaining intact lymphoid tissue architecture to support the immune response^10^,^11^,^12^,^13^. Our studies revealed that LT/LIGHT signaling played a detrimental role in Rag1^–/–^ mice defense against HSV-1 infection, which is possibly due to its role in regulating innate inflammation. LT/LIGHT signaling has been shown to induce the expression of various chemokine such as CXCL13 and CCL21, which guide or recruit immune cells to lymphoid tissue^9^,^20^,^21^. Treatment with LTβR-Ig protein could effectively alleviate several autoimmune diseases with substantially attenuated inflammation^19^,^24^,^32^. In our study, we found that the DRG was a key site where LTβR-Ig treatment inhibited HSV-1 infection and further spread to the spinal cord (Fig. 3a). LTβR-Ig treatment inhibited innate immune cell infiltration to the DRG of Rag1^–/–^ mice after HSV-1 infection on day 8 p.i (Fig. 4c). However, this reduced innate immune response did not result in an uncontrolled but restrained HSV-1 replication (Fig. 3a). We further found that LTβR-Ig treatment reduced the mRNA level of chemokines (CCL2, CXCL10 etc.) on day 6 p.i., when there is no difference of viral load or lytic associated gene ICP0 mRNA (Figs. 4a,b and 3a). This indicated that blockade of LT/LIGHT signaling by LTβR-Ig directly contributed to the reduction of inflammatory infiltration to the DRG due to a decrease of chemokine expression, followed by inhibition of viral replication. Studies have shown that control of innate inflammation by administration of anti-inflammation drugs or depletion of macrophages and neutrophils could provide neuroprotection in the HSE model^6^,^33^,^34^. Our data and others suggest that LTβR-Ig could be a candidate therapy for HSE to control viral infection and limit excessive inflammation within neuron tissue.

Overall, we have shown that LT/LIGHT signaling promotes chemokine expression and inflammatory cell infiltration, as well as HSV-1 replication in the DRG and viral spreading to the CNS, resulting in lethal inflammation in the infected host. Our data, that blockade of LT/LIGHT signaling reduced innate inflammatory cell recruitment and viral replication in the DRG, leave the intriguing possibility that infiltrating inflammatory cells and associated cytokines could drive HSV-1 replication through yet undiscovered mechanisms. In some instances, neuronal cells could be stressed by type I interferon and other cytokines to enter an apoptotic state which promotes HSV-1 reactivation and replication^35^,^36^. We propose that the positive feedback loop, from HSV-1 infection, induction of cytokines/chemokines, cell infiltration, viral replication, and inflammation, might be initiated to stimulate adaptive immune responses to promote viral clearance. In the absence of adaptive immunity, HSV-1 induced inflammatory cytokine response is not sufficient for viral control and could be maladaptive and lethal to the host. Several studies have demonstrated that type 1 IFN is less effective for HSV-1 inhibition in DRG neurons than in mitotic cells^35^,^36^. How the host inflammatory responses effect HSV-1 replication in the nervous system of immunocompromised hosts has not been well defined and should be an important aspect of future studies.

In summary, our study has uncovered a surprising and critical role of LT/LIGHT signaling in modulating innate inflammatory response to HSV-1. Understanding the role of this pathway in driving HSV-1 replication and pathology could inform the development of novel therapeutic strategies for the treatment of recurrent herpes keratitis and sporadic encephalitis in neonates and immunocompromised patients. LTβR-Ig has been shown effective in alleviating multiple inflammation disease and may be useful in treating HSV-1 associated disease.

## Methods

### Mice

Rag1-deficient C57BL/6j strain (Rag1^–/–^) were obtained from the Model Animal Research Center (Nanjing, China). LIGHT deficient (LIGHT^–/–^) mice were described previously^37^. Rag^–/–^LIGHT^–/–^ double knockout mice were generated by crossing Rag1^–/–^ with LIGHT ^–/–^mice. Mice were used at 6 to 10 weeks of age. Animal care and experiments were performed in accordance with the guidelines of the Institute of Biophysics, Chinese Academy of Sciences, using protocols approved by the Institutional Laboratory Animal Care and Use Committee.

### HSV-1 infection and analysis

HSV-1F strain was kindly provided by Dr. Thomas Kristie, LVD/NIAID/NIH, amplified in Vero cells (ATCC), collected from cell supernatant and purified through sucrose-dextran gradient centrifuge based on method reported previously^38^. 2-10 × 10^6^ pfu of HSV-1 in 40 ul PBS was subcutaneously injected into the footpad of mice. Viral titers in the footpad, DRG (L3, L4 and L5) and spinal cord were determined by plaque assay^39^. Briefly, tissues were removed and stored in DMEM (2% FBS) at -80 oC. When using, tissues were homogenized and centrifuged at 400 g for 10 min. The supernatant with serial dilutions were added onto Vero cell monolayers in 12-well tissue culture plates, and then overlaid with 0.5% methylcellulose. After 2 days of culture, plaques were visualized and counted after 0.1% crystal violate fixation.

### *In vivo* signaling and cytokine blockade

For blockade of signaling *in vivo*, 100 μg of LTβR-Ig^23^,^24^ or recombinant human TNFR-Ig (Shanghai CP Guojian Pharmaceutical Co., Ltd.) was injected i.p. on day one before (day -1) and 5 days post infection (p.i.). Irrelevant recombinant Ig was used as the control protein. Anti-LTβ (BB-F6, Biogen) or control hamster IgG was administrated 0.25 mg every 3 days from day -1. Anti-IL-6 (MP5-20F3, Bioxcell) or control Rat IgG was administrated at 0.4 mg every 3 days starting at day -1.

### Detection of cytokine

Deeply anesthetized mice were intracardially perfused with 50 ml PBS. Spinal cord and brain were homogenized and centrifuged at 4000 g for 10 min. The supernatant was collected. IL-6, MCP-1, TNF, IFN-γ, IL-10 and IL-12 in supernatant or serum were detected by mouse inflammation cytometric bead array (CBA) assay (BD Biosciences).

### Quantitative RT -PCR

RNA was extracted using RNeasy Plus Universal Mini kit (Qiagen) and then reverse transcribed into cDNA using the First Strand cDNA Synthesis Kit (Thermo Scientific). Real-time RT PCR was performed with SSoFast EvaGreen supermix (Bio-Rad) and different primer sets on StepOne Plus (Applied Biosystems). Primers used were:

β-actin- forward (F) 5′-CTG ACG GCC AGG TCA TCA CTA-3′, β-actin- reverse (R) 5′-CCG GAC TCA TCG TAC TCC TGC-3′, CCL2- F 5′-GTC CCT GTC ATG CTT CTG G-3′, CCL2- R 5′ –GCG TTA ACT GCA TCT GGC T-3′, CXCL10-F 5′-AAA TCA TCC CTG CGA GCC TAT-3′, CXCL10-R 5′-CTG CTC ATC ATT CTT TTT CAT CGT-3′, CXCL1/2-F 5′-CCA CCC GCT CGC TTC TC-3′, CXCL1/2-R 5′-CAC TGA CAG CGC AGC TCA TT-3′, ICP0-R 5′-CTG CGC TGC GAC ACC TT-3′.

The levels of gene expression were normalized to β-actin and calculated as fold change according to the 2^−*ΔΔCT*^ method.

### Flow Cytometry

Single cell suspension of lumbar DRGs (L3, L4 and L5) related to sciatic nerve were prepared as reported^39^. Mice were sacrificed and perfused with PBS. DRGs (L3, L4 and L5) were collected in 0.6 ml of collagenase type 3 (Worthington) at 3 mg/ml in RPMI (2% FBS). DRG was incubated at 37oC for 1.5 h and prepared for single cell suspension by pipetting up and down at 1 h and 1.5 h. The following antibodies were used: anti-CD45, CD11b, CD11c, Ly6c (all from Biolegend); MHCII, F4/80 (from eBioscience) and anti-CD16/32 (2.4G2). Samples were acquired on BD LSR Fortessa instruments and analyzed with FlowJo software.

### H&E staining and immunohistochemistry staining

DRG was fixed in formalin and embedded in paraffin. Tissue sections of 5μm thickness were stained with hematoxylin and eosin (H&E). For immunohistochemistry staining, tissue sections were stained with anti- mouse CD45 antibody (Biolegend). The slides were captured at 20 x magnification using Olympus camera.

### Statistical Analysis

Mean values were compared using unpaired t test or log rank (Mantel-Cox) test where appropriate. Analyses were performed using GraphPad Prism version 5.0 (Graphpad software). Statistically significant differences of *p* < 0.05, *p* < 0.01 and *p* < 0.001 are noted with *, ** and *** respectively.

## Author Contributions

Y.L., H.P. and Y.X.F. designed the experiments and analyzed the data; Y.L., K.Y. and J.G. conducted the experiments; J.G. and J.W. contributed to reagents/materials; H.P. and Y.X.F. supervised the experiments; Y.L., H.P., J.W. and Y.X.F. wrote the manuscript.

## Additional Information

**How to cite this article**: Liang, Y. *et al.* Innate lymphotoxin receptor mediated signaling promotes HSV-1 associated neuroinflammation and viral replication. *Sci. Rep.* **5**, 10406; doi: 10.1038/srep10406 (2015).

## Supplementary Material

Supplementary Information
