# Supplementary material for: Innate lymphotoxin receptor mediated signaling promotes HSV-1 associated neuroinflammation and viral replication
Source: Sci Rep. 2015 May 20;5:10406. doi: 10.1038/srep10406 (PMC4438665; doi:10.1038/srep10406)
Supplement: Supplementary Information [file srep10406-s1.pdf]

Innate lymphotoxin receptor mediated signaling promotes HSV-1 associated neuroinflammation and viral replication

Yong Liang[1,2], Kaiting Yang[1,2], Jingya Guo[1], Joanna Wroblewska[3], Yang-Xin Fu[3*] and Hua Peng[1*]

[1]Key Laboratory of Infection and Immunity, Institute of Biophysics, Chinese Academy of Sciences, Beijing 100101, China.

[2]University of Chinese Academy of Sciences, Beijing 100049, China.

[3]Department of Pathology and Committee on Immunology, University of Chicago, Chicago, Illinois 60637, USA.

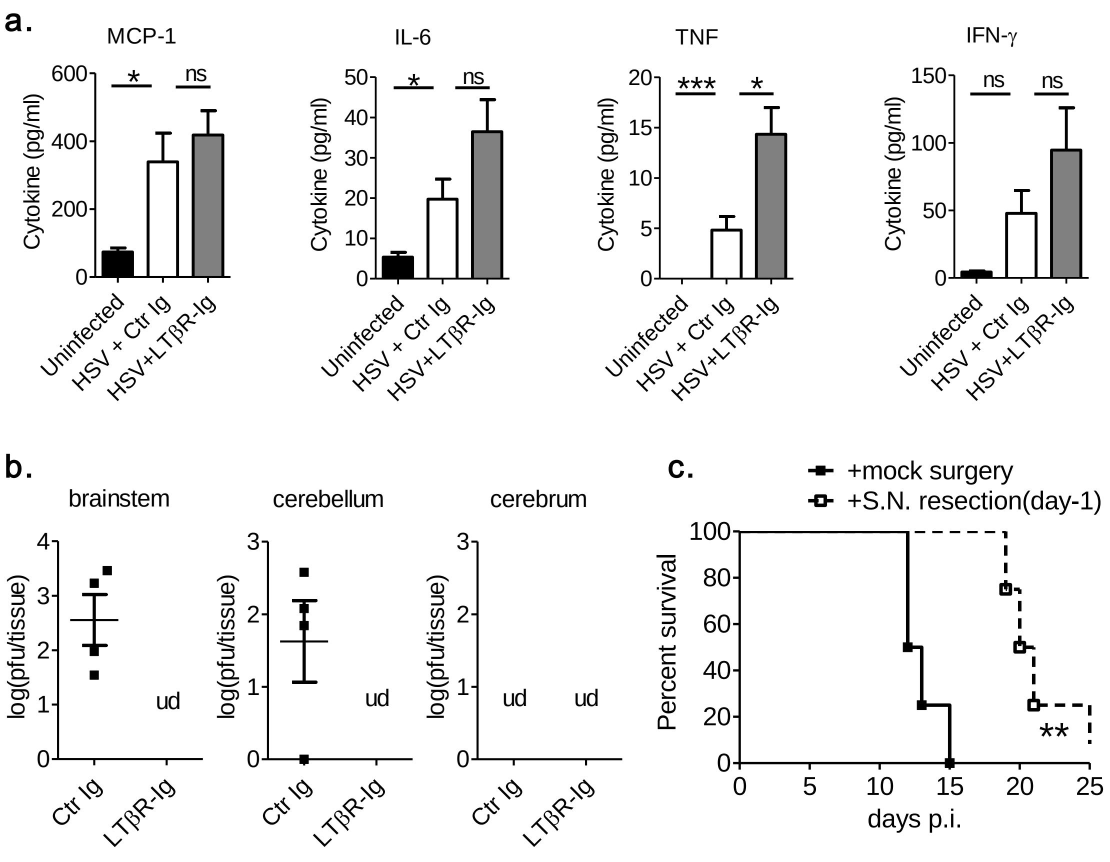

Supplemental Figure 1. (**a**) Early inflammatory cytokine profile in nervous tissue of HSV-1 infected Rag1$^{-/-}$ mice. Rag1$^{-/-}$ mice (n = 4 to 5) were subcutaneously injected with 1X10^{7} pfu of HSV-1 and treated with LTβR-Ig or control protein on day -1 and day 5 p.i.. Uninfected mice were chosen as the control group. On day 1 p.i., the level of inflammatory cytokines in serum was determined. (**b**) viral load in brain tissue. Brainstem, cerebellum and cerebrum were collected from moribund Rag1$^{-/-}$ mice (n = 4) and LTβR-Ig treated mice that had been infected on the same day and were still alive. Viral load in the homogenate of tissues was determined by plaque assay. (**c**) Temporary blocking HSV-1 spread to nervous tissue extended the survival of Rag1$^{-/-}$ mice. For mice with sciatic nerve (SN) resection, one segment of SN in midthigh of hindlimb was resected on day 1 before 2x10^{6} pfu of HSV-1 injection to ipsilateral F.P.. n = 4. Representative data are shown from two (**a** to **c**) experiments. Statistical analysis for **a.**,**b.** unpaired t test, **c.** log rank test.

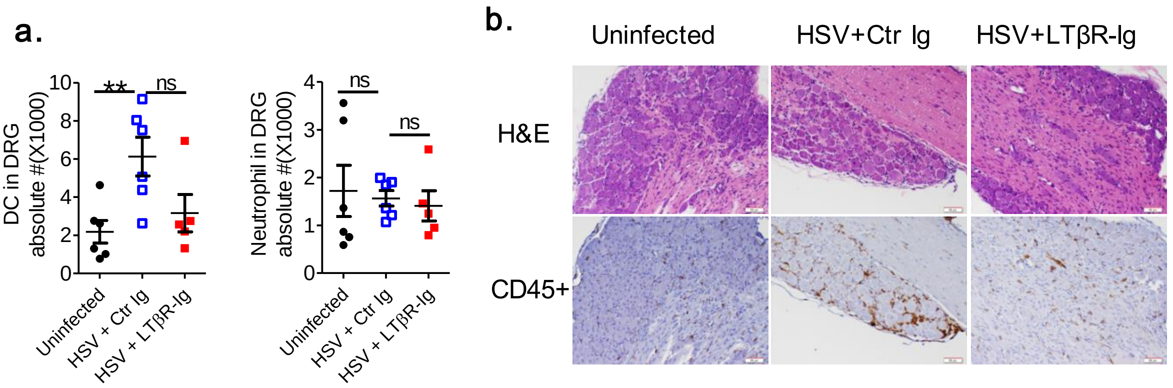

Supplemental Figure 2. LTβR-Ig treatment inhibits inflammatory cell infiltration into DRG. **(a)** samples were collected as **Figure 4c**. Gate strategy: DC ($CD45^{+}$ $Ly6G^{low}$ $MHCII^{+}$ $CD11c^{+}$), neutrophil ($CD45^{+}CD11b^{+}Ly6G^{hi}$). Data are pooled from two independent experiments, n = 5 to 6. Statistical analysis is unpaired t test. **(b)** $Rag1^{-/-}$ mice were subcutaneously injected with $2X10^{6}$ pfu of HSV-1 and treated with LTβR-Ig or control protein at day-1 and day 5 p.i.. At day 8 p.i., Histological analysis of representative DRG from indicated mice with different treatment are shown. The upper row is H&E staining, the bottom row is IHC staining for $CD45^{+}$ cell. Experiments were repeated twice.
